# Storms and silence: a case report of catatonia and paroxysmal sympathetic hyperactivity following cerebral hypoxia

**DOI:** 10.1186/s12888-020-02878-5

**Published:** 2020-09-29

**Authors:** Dallas Wolfgang Hamlin, Nuzhat Hussain, Aum Pathare

**Affiliations:** 1 Department of Psychiatry and Behavioral Health, Penn State College of Medicine, Hershey, PA USA; 2 Department of Psychiatry, Yale University School of Medicine, New Haven, CT USA

**Keywords:** Neuropsychiatry, Catatonia, Electroconvulsive therapy, Psychosomatic medicine

## Abstract

**Background:**

Delayed Post Hypoxic Leukoencephalopathy (DPHL) is a syndrome that occurs after hypoxia, and can present with a variety of neuropsychiatric symptoms, including catatonia and paroxysmal sympathetic hyperactivity (PSH). The gold standard for the treatment of catatonia is electroconvulsive therapy (ECT). However, ECT can exacerbate the paroxysms of sympathetic hyperactivity and complicate recovery from DPHL. The treatment of PSH is not well established.

**Case presentation:**

We present a case of a patient with multiple opiate overdoses who presented with altered mental status. He was diagnosed with catatonia and subsequently treated with ECT. His clinical condition worsened, and a revised diagnosis of PSH was established. The patient’s condition improved with medical management.

**Conclusion:**

This case highlights the need to distinguish between these two related symptom clusters, as the incidence of DPHL and opioid overdose related neuropsychiatric problems increase. This distinction can greatly influence the course of treatment, and the need to consider alternative treatments.

## Background

Delayed Post Hypoxic Leukoencephalopathy (DPHL) is a syndrome characterized by demyelinating injury and the acute onset of neuropsychiatric symptoms in the days to weeks following hypoxic brain injury, which can include parkinsonism, catatonia, and paroxysmal sympathetic hyperactivity (PSH) [1]. DPHL was originally described as a rare complication carbon monoxide poisoning that occurred in 3% of victims [2], however, it has been described across a range of hypoxic injuries including overdose, strangulation, and trauma [3]. It occurs on average 19 days after an initial hypoxic injury [4], and outcomes range from complete recovery to permeant disability.

Pathophysiological mechanisms proposed for DPHL have included Arylsulfatase A (ARSA) deficiency because of the histologic similarities between DPHL and metachromatic leukodystrophy, but subsequent studies showed ARSA deficiency to be neither necessary nor sufficient for the development of DPHL [4–6]. More recently, Meyer et al. (2013) proposed that the disorder is caused by injury at the time of hypoxia, but the manifestation of symptoms is delayed due to the half-replacement time of several myelin-related proteins.

One of the recognized sequelae of DHPL is the development of catatonia, which can present in the days to weeks, or even years after acquired brain injury with presentations ranging from mild to severe in nature [1, 7, 8]. While the exact pathophysiological mechanisms of catatonia remain to be elucidated, ongoing research continues to advance in describing both its structural basis as well as etiologically relevant neuroactive substances such as the brain-derived neurotrophic factor (BDNF) and antibodies against the N-methyl-D-aspartate receptor [9–11].

PSH is another entity observed in the setting of DPHL. It has been most frequently reported in patients with traumatic brain injury but has also been reported with other neurological conditions such stroke [12]. Approximately 8–33% of the patients with acquired brain injuries develop PSH. The syndrome has a striking presentation with episodes of tachycardia, hypertension, tachypnea, hyperthermia, diaphoresis and decerebrate posturing occurring in response to afferent stimulation. In most cases, the features of PSH are provoked by non-noxious stimuli or are persistent physiological responses to noxious stimuli [13, 14]. Both the onset and the duration of the episodes are variable, ranging from less than 2 weeks to many months; in some cases, residual dystonia and spasticity persist chronically after the sympathetic storms subside. Notably, PSH may clinically resemble other entities such as malignant catatonia or neuroleptic malignant syndrome and may share triggers such as neuroleptic agents [15]. However, the temporal dissociation between motor signs such as mutism, posturing, staring, immobility, rigidity and autonomic signs such as tachycardia and hypertension allow distinction between NMS and PSH.

The pathophysiology of PSH is not well defined. Suggested mechanisms have included disconnect between one or more cerebral centers from caudal excitatory centers, or disconnection of descending inhibitory pathways causing spinal circuit excitation with paroxysms, which then resolve in response to recovery of the inhibitory drivers [14]. Patients with mid brain and pontine lesions appear to be at a greater risk of PSH; as are those with lesions in the periventricular white matter, corpus callosum and deep grey nuclei.

Here, we present a case of a patient with PSH in the setting of DPHL who was initially diagnosed and treated for catatonia. The case presentation is unique in that it highlights important clinical distinctions between catatonia and PSH, and it demonstrates how appropriate management of the former is deleterious for the latter. This is especially relevant as the incidence of DPHL and opioid overdose related neuropsychiatric problems increase. The patient provided written informed consent after receiving a full description of the study and consented to the publication of this case presentation.

## Case presentation

A 25-year-old male with an opioid use disorder and multiple overdoses on heroin, presented to our hospital with acute mental status changes. A month prior to this presentation, he had been found after being down for nearly 8 h following a heroin overdose, and had required intubation and ventilator support. He subsequently underwent physical rehabilitation, and apparently recovered over the course of a month.

Soon after his return, he was noted to be confused, and was seen by an outpatient psychiatrist who started him on lurasidone for treatment of suspected mania. His confusion did not improve, after which he was brought to our ED. He was found to be disoriented, and he demonstrated involuntary jerky movements of his extremities, along with a shuffling gait. He did not display any features suggestive of mania, but presented as delirious. CBC, BMP UDS and CT head were unrevealing, and he was admitted to the internal medicine service. His cognition continued to fluctuate for the next 3 days, during which he was given haloperidol 5 mg twice for agitation and restlessness. He subsequently started demonstrating symptoms of catatonia, scoring 21 on the Bush-Francis Catatonia Rating Scale (BFCRS), with staring, grimacing, stereotypies, verbigeration, rigidity, negativism, ambitendency and perseveration.

Administration of lorazepam 2 mg IV resulted in the score dropping to 19 within 30 min, after which he was started on lorazepam 2 mg q4hrs. He had a partial response to his medication, and a decision was made to pursue ECT with a court order due to the refractory nature of his symptoms.

The initial treatment of ECT was without adverse events, and he became more interactive with his surroundings and family. However, after the second treatment, a brief period of diaphoresis, rigidity, tachycardia, tachypnea, for which was upgraded to the intensive care unit, but was sent back to the floor shortly. Two hours after the third ECT, he developed an abrupt episode of decerebrate posturing with profound diaphoresis, severe tachycardia (heart rate in 180 s), tachypnea (respiratory rate > 70), and his blood pressure went up to 180/100. He responded minimally to lorazepam and dantrolene, and was intubated due to increased work of breathing. Sedation was initially maintained with fentanyl, propofol and standing lorazepam. MRI brain without contrast showed bilateral white matter T1 hypointensities, and T2 hyperintensities involving the entire centrum semiovale. EEG showed generalized slowing without epileptiform activity. He was extubated and switched to dexmedetomidine for sedation, and unsuccessful trials of valproate and amobarbital were undertaken to wean off dexmedetomidine while treating refractory catatonia and autonomic symptoms.

Since its onset, dysautonomia had best responded to dexmedetomidine. Therefore, oral clonidine 0.1 mg TID was introduced to wean the patient from dexmedetomidine due to a shared mechanism of action. He appeared to respond well to the introduction of this agent, and clonidine was increased to a total dose of 2.1 mg/day, and propranolol was added to aid with further control of sympathetic hyperactivity. The episodes of dysautonomia did not recur with these changes, and his cognition and behavior improved in parallel with reduced sympathetic arousals. Propranolol was stopped soon after, while a taper of clonidine and lorazepam was initiated. He was discharged to a physical rehabilitation facility in 2 weeks with a plan to continue tapering the clonidine and lorazepam. Cognition continued to improve through 2 months of follow up, without return of PSH.

## Discussion and conclusion

Presently, there is no standard protocol for managing DPHL with little supporting data for any one treatment. Several case reports detail the use of hyperbaric oxygen therapy for DPHL caused by carbon monoxide poisoning, but authors of these reports note that this clinical improvement could be attributed to the natural history of the disease as opposed to a specific therapy [1, 16]. Other pharmacological agents that have been used in isolated cases of DPHL with varying degrees of success for specific symptoms. Specifically, case reports describe use of amantadine and carbidopa-L-Dopa for their dopaminergic properties, memantine and donepezil for cognitive symptoms, magnesium sulfate for its neuroprotective properties, and benzodiazepines, as well as ECT for catatonia [1, 7, 17–21]. Research has shown differential response to treatment of catatonia as a function of its etiology such as a primary psychotic disorder compared to a general medical condition, but benzodiazepines and ECT remain mainstays of therapy [22, 23]. ECT been used in in the treatment of catatonia arising from DPHL with mixed results. These from range from some clinical improvement to exacerbation of symptoms. In particular, one review noted that patient treated with ECT in the interval between hypoxic injury and the onset of DPHL had worse outcomes than individuals treated after the development of ECT. In view of this, authors from one case report recommend waiting until 1 month after the initial hypoxic injury before commencing ECT [24].

The primary aim of the treatment for PSH include avoiding triggers that promote paroxysms, preventing and mitigating excess sympathetic hyperactivity and addressing the effects of PSH on other organ systems through supportive therapy. Most paroxysms in PSH, like in our patient’s case, are responses triggered by external stimuli. This can be contrasted with dysautonomia seen in catatonia, which would not have such triggers and would instead typically have motor and autonomic symptoms together in a non-paroxysmal fashion. No pharmacologic treatment protocol is accepted as standard of care for the management of PSH, but treatment is generally directed at stabilizing vitals and supportive care. A review of the classes of drugs used for treatment and prevention of PSH are described elsewhere [13]. In clinical practice, however, patients require treatment with multiple agents to target different components of the syndrome.

Although this presentation relays important information about a clinically important syndrome at the nexus of psychiatry and neurology, certain limitations exist in this case. First, it could not be determined if the patient had an underlying psychiatric disorder prior to his initial overdose. Second, as genetic analysis was not pursued in this case, we cannot determine if the patient had a specific vulnerability to neurological insults as described in other literature. Finally, because there is a paucity of similar presentations in the literature, we cannot determine if this patient’s clinical course is characteristic of PSH in the setting of DPHL. These limitations can be addressed with further study of larger patient populations.

In conclusion, DPHL is a clinically distinct entity that arises in the days to weeks after hypoxic injury to the brain. This syndrome can include other sequelae such as malignant catatonia or paroxysmal sympathetic hyperactivity, which can have similar clinical presentations. While the standard treatment for malignant catatonia is ECT, this same treatment can provoke an exacerbation of DHPL, including PSH. The episodic nature of PSH, and its temporal dissociation from motor symptoms of catatonia is a distinguishing feature that may help differentiate these entities. This is increasingly important distinction as there is a growing population of individuals who have survived hypoxic brain injury in the context of drug overdose and are vulnerable to these distinct disorders with different management strategies.

## Data Availability

All data generated or analyzed during this study are included in this published article.

## Abbreviations

ARSA: Arylsulfatase A
CBC: Complete Blood Count
BFCRS: Bush Francis Catatonia Rating Scale
CT: Computed Tomography
BMP: Basic Metabolic Panel
DPHL: Delayed Post Hypoxic Encephalopathy
ECT: Electroconvulsive Therapy
BDNF: Brain Derived Neurotrophic Factor
PSH: Paroxysmal Sympathetic Hyperactivity
UDS: Urine Drug Screen
